# A Novel Technique of Amniotic Membrane Preparation Mimicking Limbal Epithelial Crypts Enhances the Number of Progenitor Cells upon Expansion

**DOI:** 10.3390/cells12050738

**Published:** 2023-02-24

**Authors:** Jovana Bisevac, Morten Carstens Moe, Liv Drolsum, Olav Kristianslund, Goran Petrovski, Agate Noer

**Affiliations:** 1 Center for Eye Research and Innovative Diagnostics, Department of Ophthalmology, Oslo University Hospital, P.O. Box 4956 Nydalen, 0424 Oslo, Norway; 2 Institute of Clinical Medicine, Faculty of Medicine, University of Oslo, P.O. Box 1171 Blindern, 0318 Oslo, Norway; 3 Anterior Segment of the Eye, Department of Ophthalmology, Oslo University Hospital, P.O. Box 4956 Nydalen, 0424 Oslo, Norway

**Keywords:** limbus, HAM, crypt (CLET, LESC, progenitor, differentiation, cornea, epithelial, p63α, SOX9, KRT3, KRT12)

## Abstract

We aimed to investigate whether a novel technique of human amniotic membrane (HAM) preparation that mimics the crypts in the limbus enhances the number of progenitor cells cultured ex vivo. The HAMs were sutured on polyester membrane (1) standardly, to obtain a flat HAM surface, or (2) loosely, achieving the radial folding to mimic crypts in the limbus. Immunohistochemistry was used to demonstrate a higher number of cells positive for progenitor markers p63α (37.56 ± 3.34% vs. 62.53 ± 3.32%, *p* = 0.01) and SOX9 (35.53 ± 0.96% vs. 43.23 ± 2.32%, *p* = 0.04), proliferation marker Ki-67 (8.43 ± 0.38 % vs. 22.38 ± 1.95 %, *p* = 0.002) in the crypt-like HAMs vs. flat HAMs, while no difference was found for the quiescence marker CEBPD (22.99 ± 2.96% vs. 30.49 ± 3.33 %, *p* = 0.17). Most of the cells stained negative for the corneal epithelial differentiation marker KRT3/12, and some were positive for N-cadherin in the crypt-like structures, but there was no difference in staining for E-cadherin and CX43 in crypt-like HAMs vs. flat HAMs. This novel HAM preparation method enhanced the number of progenitor cells expanded in the crypt-like HAM compared to cultures on the conventional flat HAM.

## 1. Introduction

The homeostasis of the dynamic cellular organization in the cornea mainly depends on the regenerative efficiency of the stem cells in the surrounding limbus [1]. Tissue-specific human limbal epithelial stem cells (hLESCs) residing in the limbal epithelial crypts [2] of the palisades of Vogt continuously compensate for the loss of superficial human corneal epithelial cells (hCECs) [3,4]. The insufficient compensation of diminished hCECs in the corneal epithelium due to the lack or malfunction of hLESCs leads to severe ocular surface disease or so-called limbal epithelial stem cell deficiency (LSCD) [5]. The hLESCs play an essential role in epithelial differentiation, angiogenesis, and extracellular matrix (ECM) organization [6].

Diverse therapeutic approaches have been used to treat both monocular and binocular LSCD. However, cultivated limbal epithelial stem cell transplantation (CLET) of expanded autologous limbal tissue seems to be the most common method for monocular LSCD [7,8]. The CLET procedure is based on isolating the limbal biopsy from the contralateral eye and treatment with a proteolytic enzyme to digest the surrounding ECM, which helps the hLESCs get released and migrate from the niche. Furthermore, the digested limbal tissue or single isolated hLESCs are harvested ex vivo in a medium containing stem-cell-supporting growth factors and supplements, achieving cell expansion and graft tissue synthesis [9]. Upon transplantation, hLESCs reside on the damaged corneal-limbal tissue, re-creating the limbal stem cell niche that allows epithelial regeneration. 

Following the existing standard protocols, the reported success rate of CLET varies. A favorable morphological outcome implying stable, intact, completely epithelized and avascular corneal surface is reported as 46.7% to 80.9%, whereas success as a functional outcome such as visual acuity varies from 60.5% to 78.7% [10,11,12,13,14,15,16,17,18,19,20]. Successful transplantation is directly dependent on the graft tissue quality and the percentage of hLESCs/early progenitor cells in the graft. For successful transplantation, at least 3% of the cells in the expanded cell culture must express the p63 marker [11]. Therefore, establishing a protocol that provides a high percentage of the hLESCs/early progenitor cells in the transplantation graft is of high importance.

Human amniotic membrane (HAM) has proven to be a very efficient therapeutic tool in many ocular surface diseases, supporting wound healing and regeneration while suppressing inflammation [21,22], angiogenesis [23], and fibrosis [24,25,26], and it possesses anti-microbial features [27,28]. It is used for corneal epithelial regeneration, conjunctival reconstruction, glaucoma interventions, and the treatment of corneal melting and perforations. Importantly, it is one of the most used carriers for the ex vivo expansion of hLESCs [26,29,30]. HAM contains stem cell niche factors that support maintenance [31]. Generally, such maintenance depends on the inhabitance of the stem cells in a specific niche that allows their anchoring and communication with supporting cells, the release of specific growth factors and cell cycle molecules, and the involvement of evolutionary conserved molecular pathways. Within the niche, the stem cells undergo symmetric or asymmetric division to transient amplifying cells (TACs) that leave the environment and become functionally mature corneal cells [32].

It is known that the physical cues of the cellular environment guide stem cell fate [33,34,35]. It is also suggested that biomechanical changes in the limbal stromal niche affect hLESCs fate [36]. No less importantly, mechanical and environmental changes in the corneal tissue have implications for some corneal diseases and pathologies [37]. We hereby present a novel suturing preparation technique that causes the three-dimensional (3D) radial folding of the HAM, mimicking crypt-like formations. The novel approach may allow the hLESCs, upon limbal biopsy expansion and cultivation ex vivo, to reside in the undulated crypts of HAM. This may potentially maintain the putative characteristics of the expanded hLESCs and thus ensure a higher quality of the expanded graft tissue compared to the conventional state-of-the-art method. Therefore, we aimed to compare the progenitor/differentiation state of the cells cultivated in the crypt-like HAMs vs. the cells cultivated on the flat-like HAMs.

## 2. Materials and Methods

The Regional Committee for Medical and Health Research Ethics in South-Eastern Norway (No 2017/418) approved tissue harvesting and laboratory procedures, and all tissue collections complied with the Guidelines of the Helsinki Declaration. Unless stated otherwise, all reagents were purchased from Merck (Darmstadt, Germany).

### 2.1. Human Amniotic Membrane (HAM)

A Placenta was collected after a scheduled cesarian section from a full-term pregnancy. Informed consent and institutional board review approval had previously been obtained from the patient. According to the standard protocol, the placenta was immediately transported in a sterile container and further processed under sterile conditions [26]. Proper washing with 0.9% NaCl (Fresenius Kabi AB, Uppsala, Sweden) or 0.9% NaCl containing 100 U/mL Penicillin, 100 μg/mL Streptomycin (P4333), and 2.5 μg/mL Amphotericin B (A2942) was repeatedly performed. The HAM was then separated from the chorion by blunt dissection, washed out from residual blood, and transferred onto a nitrocellulose filter carrier, pore size 0.45 μm (111306-47-CAN, Sartorius, Gottingen, Germany) with the epithelial side up, then divided into 3 × 3 cm and 5 × 5 cm pieces. HAM pieces were cryopreserved in 50% glycerol, 48.5% DMEM/F12 (31331028, Invitrogen, Carlsbad, CA, USA), 100 U/mL Penicillin, 100 μg/mL Streptomycin and 2.5 μg/mL Amphotericin B and stored at −80 °C.

### 2.2. HAM Preparation for hLESC Expansion and Cultivation

**Figure 1:**
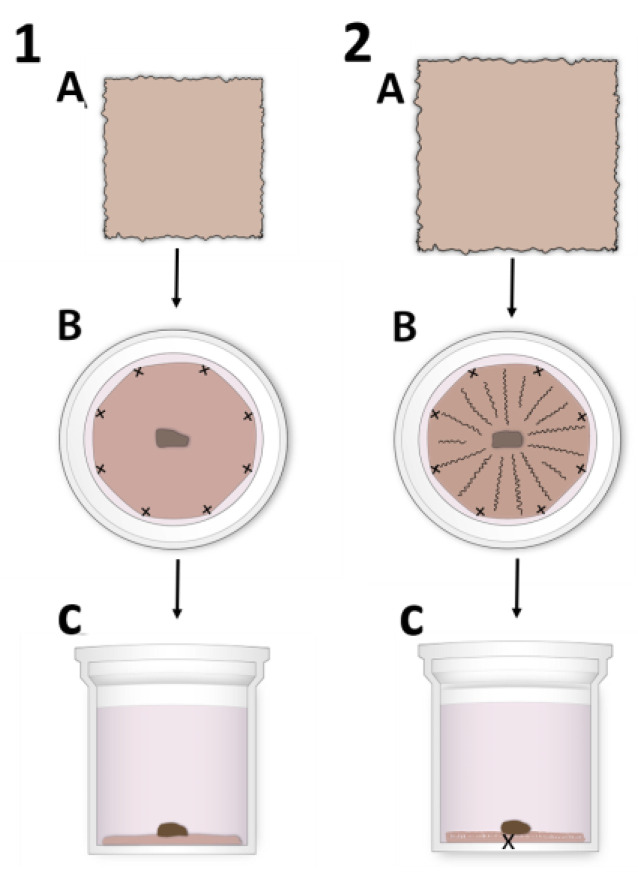
Illustration of the different human amniotic membrane (HAM) suturing techniques for human limbal epithelial stem cell (hLESC) expansion and harvesting. (1) The standard preparation procedure using (**A**) 3 × 3 cm HAM tissue that is stretched over and sutured to the polyester membrane of the NetwellTM insert by the individual sutures ((**B**): horizontal section, (**C**): vertical section of the NetwellTM insert). The limbal biopsy is placed centrally on top of the HAM with the epithelial side down. (2) The novel preparation technique using (**A**) HAM tissue 4 × 4 cm or 5 × 5 cm in size that is placed on the top of the membrane and sutured by individual sutures (**B**,**C**). The HAM is loosely attached to the membrane surface, forming crypt-like protrusions. In addition, the individual/single suture is placed centrally to obtain the HAM folding and hold it tight to the membrane.

Before use, HAMs were thawed, warmed to room temperature, and washed three times with a medium containing DMEM/F12, 100 U/mL Penicillin, and 100 μg/mL Streptomycin. Thereafter, the HAMs were placed on polyester membrane Netwell^TM^ inserts (3479, Corning Inc., New York, NY, USA) 24 mm in diameter, with the epithelial side up by two different techniques (Figure 1): (1) HAMs 3 × 3 cm (Figure 1.1A) in size were peeled from the nitrocellulose filter paper. Further, HAMs were placed and stretched on the top of the polyester membrane and sutured by the eight individual sutures (Figure 1.1B) near the edge of the polyester membrane. The HAMs were then tightly stretched on the top of the membrane, making a flat surface (Figure 1.1C). Excess HAM tissue that remained at the edge of the polyester membrane was carefully removed with a disposable sterile scalpel. (2) The other approach was to use HAMs 5 × 5 cm in size (Figure 1.2A) placed on top of the membrane and sutured so that the HAMs were loosely attached. HAMs were sutured by individual sutures (Figure 1.2B). In addition, an individual suture was placed in the center of the HAM/polyester membrane to obtain the folding of the HAM and to keep it in close contact with the membrane (Figure 1.2C). The excessive HAM tissue at the edges was again removed accordingly. The sutured HAMs on the Netwell^TM^ inserts were immersed in DMEM/F12 medium containing 100 U/mL Penicillin and 100 μg/mL Streptomycin and kept at 37 °C, 5% CO_2_, and 95% air overnight to obtain HAM free of any glycerol remains.

### 2.3. Limbal Biopsies and Human LESC Harvesting

Following corneal transplantation, the remaining human corneal-scleral rings from three donors (n = 3) were divided into twelve limbal biopsies of equal size and thoroughly washed with DMEM/F12 medium containing 100 U/mL Penicillin and 100 μg/mL Streptomycin. The biopsies were treated with neutral protease and Dispase II (2.4 U/mL 4942078001, Roche Diagnostics, Mannheim, Germany) for 10 min at 37 °C. The dissociation process was blocked using Fetal Bovine Serum (FBS, F2442). The limbal biopsies were then placed centrally on the top of the HAMs, with the epithelial side down and submerged in a standardly used complex medium (COM). COM consisted of DMEM/F12, Penicillin (100 U/mL), Streptomycin (100 μg/mL), Amphotericin B (2.5 μg/mL), human epidermal growth factor (2 ng/mL, E9644), insulin (5 μg/mL), sodium selenite (5 ng/mL) and transferrin (5 μg/mL, l1884), cholera toxin A subunit from *Vibrio cholerae* (30 ng/mL, C8180), hydrocortisone (0.03 μg/mL, H0888), 5% FBS, and 0.5% dimethyl sulfoxide (DMSO, D2650). After 2 h of incubation, the attached limbal biopsies were completely covered by COM. Further, the limbal biopsies and outgrowing LESCs were harvested and incubated at 37 °C with 5% CO_2_, and 95% air for the following three weeks. The culture medium was changed every three days.

### 2.4. Immunohistochemistry (IHC) and Immunofluorescence Microscopy

Limbal biopsies with hLESCs cultured on HAMs were cut from the Netwell^TM^ inserts. Samples were fixed in 4% formalin overnight at 4 °C and then processed in dehydrated graded alcohol series of 70% (10–15 min), 80% (10–15 min), 96% (2 × 10 min), and 100% ethanol (2 × 10 min) before having xylene added (3 × 10 min) and being washed with melted paraffin (3 × 10 min) and then embedded in paraffin for immunohistochemistry (IHC).

Paraffinized tissue was cut into 3–4 μm thick sections using an automated microtome (HM 355S, Thermo Fisher Scientific, Waltham, MA, USA) and attached to histological slides. Deparaffinization was performed in xylene (2 × 10 min), then rehydration was performed by sinking in 100%, 96%, and 70% ethanol, and then distilled water. 

Hematoxylin & Eosin (H&E) staining was primarily performed. Slides were immersed in Mayers hematoxylin plus solution (01825, Histolab, Askim, Sweden) for 10 min and then rinsed with distilled water (10 min) followed by eosin staining (10 min), and then they were rehydrated in upgraded alcohol series of 70%, 96%, 100% ethanol, and xylene. Slides were further mounted using Pertex (00840, Histolab) mounting medium.

For IHC, heat-induced antigen retrieval was performed in a microwave for 5 min at 900W and 15 min at 600W in a citrate buffer (pH 6, C9999) or by PT module (LabVision, Fremont, CA, USA). Blocking of non-specific binding sites with 5% Bovine Serum Albumin (BSA, A9418) dissolved in Dulbecco’s Phosphate Buffered Saline (DPBS, 14190-144, Thermo Fisher Scientific) was conducted for 20 min. Further, slides were stained with primary antibodies diluted in 1% BSA for 1 h. Slides were stained using antibodies for the following progenitor markers: tumor protein p63 alpha (p63α, rabbit polyclonal, 1:200 dilution, 4892S, Cell Signaling, Beverly, MA, USA), SRY-Box Transcription Factor 9 (SOX9, 82630, Cell Signaling, rabbit monoclonal, 1:200), quiescence marker: CCAAT/enhancer-binding protein delta (CEBPD, rabbit polyclonal, 1:200 dilution, ab198320, Abcam, Cambridge, UK), and proliferation marker Ki-67 (rabbit monoclonal, 1:200, RM-9106-S, Thermo Scientific) and the following differentiation markers: cytokeratin 3/12 (KRT3/12, mouse monoclonal, 1:100 dilution, 08691431, MP biomedicals, Santa Ana, CA, USA) and connexin-43 (CX43, rabbit polyclonal, 1:300, C6219). Then, the slides were thoroughly washed three times for 5 min with PBS-tween buffer (28352, Thermo Fisher Scientific). Incubation was continued with the appropriate animal type of secondary antibody: Cy3^®^ goat anti-rabbit IgG (rabbit monoclonal, 1:500 dilution, A10520, Abcam) for samples stained with p63α, CEBPD, SOX9, Ki67, and Alexa Fluor^®^ 488 donkey anti-mouse IgG (1:500 dilution, mouse monoclonal, 21202, Abcam) for samples stained with KRT3/12, and Alexa Fluor^®^ 488 donkey anti-rabbit IgG (1:500 dilution, rabbit monoclonal, A21206, Abcam) for antibody staining CX43. The secondary antibody was incubated for 45 min and washed three times for 5 min. Nuclear staining was performed using a 4′,6-daminidino-2-phenylindole (DAPI) mounting solution (P36931, Life technologies corporation, Carlsbad, CA, USA).

Further, LabVision Autostainer 360 (Lab Vision Corporation, VT) was used for staining with antibodies against adherent junction molecules such as E-cadherin (CDH1, mouse monoclonal, 1:50 dilution, n1620, DakoCytomation, Santa Clara, CA, USA) and N-cadherin (CDH2, mouse monoclonal, 1:100 dilution, m3613, DakoCytomation). Visualization was done using the standard peroxidase technique (UltravisionOne HRP system, Thermo Fisher Scientific). Primary antibody binding to an expressed antigen was recognized by a secondary antibody conjugated with peroxidase-labeled polymer with diaminobenzidine (DAB). Each staining was performed at least three times, and each sample was tested in triplicate. Negative and positive controls were performed simultaneously for all antibodies. All antibodies used for IHC in this study are summarized in Supplementary Information Table S1.

Bright-field images of H&E and DAB-stained samples were taken by a ZEISS Axio Observer Z1 microscope (ZEISS, Oberkochen, Germany). Fluorescence was recorded by a ZEISS Axio Imager M1 fluorescence microscope (ZEISS). Three independent individuals used Image J software and counted nuclear antibody positivity (p63α, CEBPD, SOX9, and Ki67). 

### 2.5. Statistical Analysis

The technical replicates from the same donor and group of three donors of the hLESC harvested in two different conditions were averaged as a percentage mean ± standard error of the mean (SEM). Prism 8.3.0 (GraphPad, San Diego, CA, USA) was used for statistical analysis. The data were counted and analyzed by two different methods: in percentages representing the ratio of the number of cells positive for a specific marker and the total number of cells (DAPI positivity), or as the number of cells positive for a specific marker per mm^2^. Further, data were tested for normal distribution (Shapiro–Wilk test), and the difference was tested using an unpaired two-sample t-test. The significance level *p* ≤ 0.05 was counted as significant.

## 3. Results

### 3.1. Epithelial and Basement Membrane (BM) Morphology in Corneal-limbal Tissue and Consequent Localization of hLESCs 

The distribution of hLESCs was examined in the different BM compartments of the human corneal-limbal tissue in situ and compared to the hLESCs cultured on conventionally flat and alternatively, HAM sutured in a radial pattern, mimicking limbal crypts ex vivo (Figure 1).

**Figure 2:**
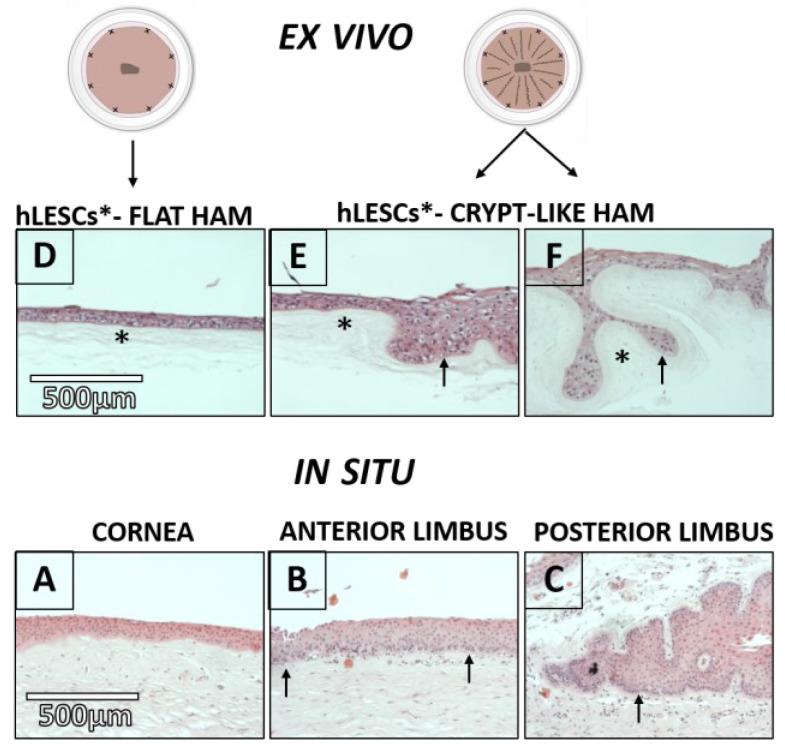
Morphology of the human epithelium in the cornea and limbus in situ compared to ex vivo expanded human limbal epithelial stem cells (hLESCs) on the human amniotic membrane (HAM). (1) Presentation of human epithelium in (**A**) cornea, (**B**) anterior limbus, and (**C**) posterior limbus and limbal epithelial crypts. The hLESC/early progenitor cells are present in the basal epithelial layer of the anterior limbus (arrow) and even more so in the posterior limbus/limbal epithelial crypts (arrow). (2) Ex vivo hLESC cultures on the (**D**) flat HAM, (**E**) flat and ((**E**), arrow) undulated HAM, and (**F**) undulated and looped ((**F**), arrow) HAM compartments. HAM is marked with a black star (*). Scale bars are the same for all images in the same row.

The human corneal epithelium had 5–7 layers on the flat BM and an avascular Bowman’s layer (Figure 2A). The anterior limbus contained 7–10 epithelial layers on the irregular BM and vascularized stroma underneath (Figure 2B). The posterior limbal epithelium was attached to the undulated BM and limbal epithelial crypts that were placed deeper and were mainly surrounded by the limbal stroma (Figure 2C). The hLESCs were smaller in size, with a high nucleo-cytoplasmic (N-: C) ratio, and could be randomly detected in the basal epithelial layer of the anterior limbus (Figure 2B, black arrows). However, the hLESCs seemed to be more present and densely packed in the basal layer of the posterior limbus and limbal epithelial crypts (Figure 2C, black arrow).

### 3.2. Morphology of the hLESC Cultures Expanded on Conventional, Flat-sutured HAMs vs. hLESC Cultures Expanded on the Novel, Radially-sutured HAMs

HAMs sutured by the novel radial suture technique comprised of flat and crypt-like areas. Furthermore, the crypts of the HAMs sutured by the novel radial suture technique consisted of (1) undulated HAM areas with the opened surface (Figure 2E) and (2) looped HAM areas that appeared to be almost closed (Figure 2F, black asterisk). The multi-layering of the epithelial cells was noted in ex vivo expanded hLESC cultures lying on the undulated and looped HAMs compared to cultures lying on the flat HAM (Figure 2E,F, black arrows vs. Figure 2D). A higher presence of columnar-like epithelial cells was noted in the cultures harvested on the undulated (Figure 2E) and looped HAMs (Figure 2F) compared to cultures harvested on the flat HAM (Figure 2D). The polygonal and squamous cells were found in the middle and superficial layers of the hLESC cultures on the flat HAMs (Figure 2D). These polygonal and squamous cells appeared to be less present in the hLESC cultures in the crypt-like HAM compartments. 

To better understand the structural differences in cultivated tissue on flat and crypt-like HAMs, we aimed to compare the marker fingerprint of cultures growing on flat and looped-like HAMs, as these are two morphologically distinct settings. 

### 3.3. Distribution of the Progenitor Markers In Situ Versus In Vitro Study Conditions

The progenitor marker p63α was found in some of the cells of the basal and suprabasal layers of the cultures expanded on the flat and undulated HAMs (Figure 3.1A,B).

**Figure 5:**
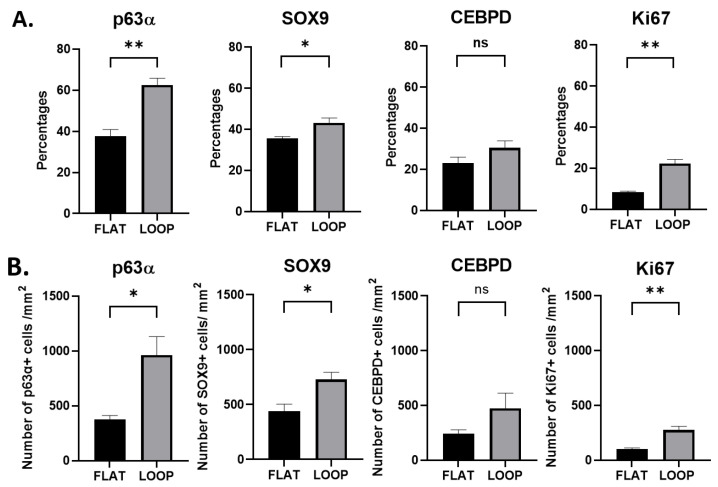
Comparison of progenitor, quiescence, and proliferation marker positivity in ex vivo expanded hLESCs cultured on flat and crypt-like HAMs. (**A**) Comparison of p63α, SOX9, CEBPD, and Ki-67 percentage positivity in hLESCs harvested on flat and looped HAMs. (**B**) In addition, the comparison of total cell number positive for specific markers per square millimeter was determined. All data from three donors (n = 3) are presented as mean ± SEM, * *p* < 0.05, ** *p* < 0.01, ns = not significant.

However, the HAM loops contained a statistically higher number of p63α-positive hLESCs (Figure 3.1C) than cultures on the flat HAM, quantified as percentages: p63α vs. DAPI positivity ratio (flat vs. loop, 37.56 ± 3.34% vs. 62.53 ± 3.32%, *p* = 0.01, Figure 5A) or as a total number per mm^2^ (377.8 ± 34.17 vs. 962.9 ± 167.2, *p* = 0.03, Figure 5B). Regarding the epithelium in the corneal-limbal tissue, p63α was not found in any of the cells of the corneal epithelium in situ (Figure 3.2A) but was identified in some cells of the basal and suprabasal layers of the anterior limbus (Figure 3.2B, arrow). The posterior limbal epithelium with undulated BM was enriched with p63α-positive cells in the basal and suprabasal layers (Figure 3.2C). 

Basal and suprabasal cells in the cultures expanded on flat (Figure 3.1D), undulated (Figure 3.1E), and looped (Figure 3.1F) HAMs expressed the SOX9 progenitor marker. However, the SOX9 progenitor marker positivity was significantly higher in the cultures expanded on crypt-like HAMs forming loops than in the cultures growing on flat HAMs, quantified as percentages (35.53 ± 0.96% vs. 43.23 ± 2.32%, *p* = 0.04, Figure 5A) or as a total number per mm^2^ (442.3 ± 62.31 vs. 728.1 ± 65.97, *p* = 0.03, Figure 5B). In situ, the progenitor marker SOX9 was exclusive for the limbal basal epithelium (Figure 3.2E). In particular, the limbal epithelial crypts appeared to be enriched for this marker (Figure 3.2F). 

### 3.4. Expression Profile of the Proliferation and Quiescence Markers in the Corneal-limbal Epithelial Tissue Versus In Vitro Study Conditions 

The expression distribution of the CEBPD marker was similar to p63α and present in the cultures expanded on flat (Figure 4.1A), undulated (Figure 4.1B), and looped HAMs (Figure 4.1C)—mainly in basal and suprabasal layers. 

In addition, CEBPD was found in the basal epithelial cells of both tissues in situ, the cornea (Figure 4.2D) and the anterior and posterior limbus (Figure 4.2B,C), accordingly. However, no statistical significance was noted in the number of CEBPD-positive cells expanded on HAM loops compared to cells expanded on flat HAMs, quantified as percentages (22.99 ± 2.96% vs. 30.49 ± 3.33 %, *p* = 0.17, Figure 5A) or as a total number per mm^2^ (243.00 ± 35.19 vs. 474.1 ± 138.5, *p* = 0.18, Figure 5B).

Many of the hLESCs expanded on a flat (Figure 4.1D, white arrow), undulated (Figure 4.1E), and looped HAM (Figure 4.1F) were found in the proliferation state. However, some sectors of the epithelial tissue on the flat HAM contained no Ki-67-positive cells (Figure 4.1D), while sections of the epithelial tissue on the undulated (Figure 4.1E) and/or looped HAM (Figure 4.1F) persistently maintained Ki-67-positive cells. Proliferation was significantly higher in cultures expanded on looped HAMs compared to the cultures on flat HAMs, quantified as percentages (8.43 ± 0.38 % vs. 22.38 ± 1.95 %, *p* = 0.002, Figure 5A) or as a total number per mm^2^ (100.7 ± 10.69 vs. 276.2 ± 33.34, *p* = 0.01, Figure 5B). For comparison to the in situ state, proliferation marker Ki-67 was sporadically found in the suprabasal cells of the anterior (Figure 4.2E) and posterior (Figure 4.2F) limbal epithelium, whereas it was absent in the central corneal epithelium, and only sparsely present in some basal cells of the posterior cornea (Figure 4.2D). 

### 3.5. Differentiation Marker profile in the Epithelium of the Corneal-limbal Tissue, and hLESC Cultures on the Flat and Crypt-like HAMs 

CX43 was uniformly distributed in all ex vivo expanded cells (Figure 6.1A–C), a finding similar to the CX43 pattern in the corneal epithelium in situ (Figure 6.2A). However, some basal cells in the anterior (Figure 6.2B) and posterior limbal epithelium and limbal epithelial crypts (Figure 6.2C) appeared to lack the CX43 marker.

In the expanded hLESC cultures growing on flat HAMs, the differentiation marker KRT3/12 was present in the polygonal and squamous cells, mainly in the middle and top layers (Figure 6.1D). Less KRT3/12 presence could be noted in the cultures expanded on undulated (Figure 6.1E) and loop HAMs (Figure 6.1F), whereas this marker was almost absent in cells growing in small HAM loops. Regarding the corneal-limbal tissue, KRT3/12 was present in all corneal epithelial cells (Figure 6.2D). In the limbal epithelium, the majority of the cells were stained positive for the KRT3/12, whereas the cells in the lowest layers attached to the BM were devoid of this marker (Figure 6.2E,F). 

### 3.6. Presentation of Cell Adhesion Molecules in the Corneal-Limbal Epithelium and Expanded hLESC Cultures on Flat and Crypt-like HAMs 

The transmembrane protein E-cadherin was present in most of the ex vivo expanded epithelial cells (Figure 7.1A–C). The same applied to the corneal-limbal tissue in situ (Figure 7.2A–C). 

In hLESC cultures, N-cadherin was present in a few cells of the basal layer on the flat HAMs (Figure 7.1D). On the other side, more cells in the basal layer of the crypt-like HAMs seemed to express N-cadherin since the surface of the basal layer of the cultivated tissue appeared enlarged in those crypts compared to the cultures on the flat HAMs (Figure 7.1E,F). N-cadherin was exclusive for the limbal basal epithelium in situ. Only a few basal cells in the epithelium of the anterior limbus expressed N-cadherin (Figure 7.2E). In contrast, almost all cells of the limbal basal epithelium in the posterior limbus expressed N-cadherin (Figure 7.2F). 

## 4. Discussion

Different techniques of HAM suturing to the corneal surface have been used thus far. A HAM can be sutured as a graft (inlay) or as a patch (overlay). While used as a graft, a HAM is placed on the defect with the stromal face down and acts as a BM, allowing the epithelium to proliferate and regenerate over it. It can be used as a single or multilayered graft with a *lamellar sac, filling, or roll-filling technique,* mostly depending on the depth of the corneal defect. The patch technique is mostly used for epithelial defects without perforations. The epithelial side of the HAM is placed down towards the defect, and the HAM serves as a biological compressive bandage [29,38]. Graft alone or as part of a sandwich technique, which is a combination of both graft and patch techniques, has been standardly used for HAMs carrying cultivated hLESCs and limbal explants [29]. However, this is the first study to propose the manipulation of a HAM prior to the expansion of the hLESCs to ensure a better quality of the transplanted tissue as an adjuvant technique to the previously used HAM suturing techniques. 

Stem cell niches vary in size and functional organization in mammals [39]. Stem cells can be found as individual structures under the BM of the skeletal muscle [40], or grouped as epithelial stem cells in the hair follicle bulges [41] and neural stem cells in the forebrain subventricular zone in mammals [42]. In this study, we provided a more optimal microenvironment for the expansion of hLESCs ex vivo by mimicking the BM folding in niches residing in the posterior limbus and limbal epithelial crypts. Generally, the maintenance of stem cells, including hLESCs, depends on a functional niche characteristic. These niches provide cell anchoring, mechanical protection, communication with underlying stroma and vasculature, the release of specific growth factors, cell cycle molecules, and the involvement of evolutionary conserved molecular pathways. Such 3D microenvironments allow the stem cells to hold the quiescence, maintain stemness, and undergo asymmetric or symmetric proliferation when needed [32].

Our study supports earlier findings that most hLESC/early progenitor cells reside on the bottom of the limbal epithelial crypts, which are deep epithelial protrusions directly surrounded by a loose stromal matrix [43]. When an epithelial stem cell niche is established along the stiff BM, it maintains its regular morphology. However, when the epithelial stem cell niche forms along a flexible and extensible BM, it may arrange in the form of finger-like protrusions, enabling a higher surface for stem cells to allocate, thus providing the protection and preservation of the putative stem cell characteristics [44]. Stem cell progenies acquire differentiation properties by leaving the stem cell niche towards the more rigid and flat ground, such as the Bowman membrane, to eventually terminally differentiate and undergo apoptosis (26). A HAM is a desirable elastic and adaptable scaffold for creating 3D protrusions that can physically mimic limbal crypts ex vivo. It is a widely available natural semi-transparent and permeable membrane. Its mechanical and functional characteristics are desirable for the migration, adhesion, and growth of epithelial cells on the ocular surface. It possesses high elasticity, low stiffness, and high tensile strength properties [45], and it also resembles the cornea and conjunctiva in regards to the collagen arrangement [46,47]. The stiffness should be similar between flat vs. crypt-like HAMs, as we used pieces from the same donor. Even though there might be some local differences in stiffness within the same HAM, we used nine pieces of both flat and crypt-like HAM, and all the pieces showed significant changes related to the suturing method. There was only one extra suture on the crypt-like vs. flat HAMs to enable the folding, so this should not have affected the overall stiffness of the crypt-like vs. flat HAMs.

Functionally, HAM is immunotolerant and has low antigenicity, even though some immunomodulatory effects have been reported; it has an anti-fibrotic impact, mainly due to the TGF-β inhibition. It secretes a wide range of growth factors, such as EGF, bFGF, HGF, KGF and KGF receptors, TGFα, and TGFβ 1,2,3 isoforms, sharing some common features with stem cell niche composition [48]. However, not all HAM properties seem beneficial for stem cell maintenance. An intact HAM promotes the epithelial differentiation of explanted limbal cultures. Therefore, removing the epithelium from the HAM upon preparation has been used by some authors to maintain progenitor properties, postpone differentiation, and thus, improve the quality of the explanted tissue [49]. Also, not all of the cells expanded on a HAM have the features of hLESCs or early progenies. As previously shown, most of the hLESC/progenies are positioned in the basal epithelial cell layers—the ones attached to the HAM. In contrast, the cells in the upper/superficial layers exhibit more differentiation properties [50,51,52]. With our novel suturing technique, we aimed to enlarge the surface area of the HAM, and hence enlarge the number of cells in the basal layer attached to the HAM, maintaining the more undifferentiated state. In addition, the expanded epithelial tissue appeared multilayered in the crypt-like HAMs, and it contained a higher number of columnar-like cells, likely indicating a higher proliferation rate. The novel suturing technique would also increase the supply of the stem-cell-supporting molecules secreted by the HAM.

HAM is widely implemented in tissue engineering and regenerative medicine [38,53]. However, its limited chemical and physical features, and the high cost of preserving it in a fresh condition, caused the urgency for new solutions [54]. HAM has, so far, undergone additional adjustments to upgrade its properties for easier manipulation, duration, and utilization, and for higher resistance to microbes and broader applications, among others, in ocular surface reconstruction [38,45,53,55,56,57,58,59]. For instance, AM can be used as a constituent of various composite scaffolds, in a form of extract, and as a hydrogel [55,60,61]. Regarding the attempts of HAM modification for successful LESC transplantation, decellularized AM (dAM) conjugated with an electrospun polymer nanofiber mesh promoted LESC proliferation and adhesion in a rabbit model [62,63]. An amniotic membrane in a form of extract (AME) and eye drops proved beneficial for the treatment of ocular surface disorders, injuries, and the in vivo cultivation of hLESCs [64,65,66,67,68]. In addition, AME, as an animal-free product, was suggested as a suitable replacement for FBS upon LSC transplantation to avoid the risk of disease transmission and accumulation of bovine antigens [68]. However, all the above-modified HAM methods require very complex processing or serve only as adjuvant therapy. Therefore, we present an easy-handling, widely available, and inexpensive method of HAM manipulation prior to hLESC expansion.

As previously mentioned, the successful long-term restoration of the corneal epithelium after CLET requires more than 3% of the cells in the transplanted graft to be p63 positive [11]. In our study, cells growing on either the flat or crypt-like HAMs were enriched with the p63α marker. However, we found a significantly larger number of cells positive for p63α in the looped regions of the crypt-like HAMs, compared to the cells growing on flat HAMs. Initially, the whole tumor protein p63 was perceived as a specific marker for hLESCs [69]. Later studies discovered ΔNp63α to be more distinct for hLESCs and for early progenies residing in the limbal basal epithelium, while other p63 isoforms were detected in the suprabasal layers of the limbus and cornea, playing a role in corneal differentiation [70,71,72]. Indeed, in our samples, p63α stained the particular cells in the basal limbal epithelium, while staining in the non-limbal cornea was absent.

Significantly higher cell turnover was present in the cultures on the crypt-like HAMs compared to the cultures growing on flat HAMs, indicating the presence of cells with intense proliferation, such as early progenies/TACs. Compared to the in situ state, proliferation appeared to be much lower in corneal-limbal tissue, and it was noted in a few suprabasal cells of the anterior and posterior limbus and some cells in the posterior cornea as previously described [73].

The CEBPD marker was not significantly more abundant in the cultures growing on crypt-like HAMs compared to those on flat-like HAMs. CEBPD is a quiescence marker that controls the cell cycle and inhibits the proliferation of hLESCs in ex vivo cultures [74]. Since proliferation was significantly higher in ex vivo cultures growing on crypt-like HAMs compared to those growing on flat-like HAM, we expected these cells not to be positive for CEBPD; hLESCs do not co-express CEBPD with the Ki-67 marker in the limbus. In addition, CEBPD-positive hLESCs that co-express the ΔNp63α marker in the basal limbal epithelium in situ are the ones considered quiescent [74]. However, it seems that the CEBPD marker is not specific for hLESCs in situ, since it is also found in the cells of the basal corneal epithelium in our samples.

The transcriptional factor SOX9 plays diverse roles in the embryonal and adult development of mammals as well as in stem cell maintenance. This marker was upregulated in the cultures on crypt-like HAMs compared to those on flat-like HAMs. Its nuclear localization in TACs is essential for proliferation upon wound healing. However, SOX9 is particularly involved in the proliferation and differentiation steps of early progenies derived from hLESCs, but not in the terminal differentiation, which explains why SOX9 is absent in the cornea [75]. This finding contributes to our conclusion that crypt-like HAMs contain numerous cells that are positive for SOX9 and are thus in a more undifferentiated state.

We found no difference in the presence of the CX43 transmembrane protein between the cultures on crypt-like HAM and flat HAM. CX43 is a protein involved in the communication between mammalian cells through diverse mechanisms [76]. Constitutionally, CX43 is present in the corneal epithelium and all suprabasal epithelial layers in the limbus, whereas it is absent in some cells of the basal limbal layer [77,78]. A similar pattern of expression of CX43 applies to expanded cultures on the flat HAMs [79]. The absence of cell interaction may be one of the mechanisms for maintaining stemness and the quiescence state [77]. Thus, according to some authors, CX43 positivity in cells distinguishes the hLESCs from the TACs/early progenies in vivo. Since most of the cells in the basal and suprabasal layers of the cultivated epithelial tissue were CX43 positive, it seems that a very small cell fraction remains in a quiescent state ex vivo.

Cytokeratin 3 (KRT3) and cytokeratin 12 (KRT12) are cornea-specific intermediate filaments, hallmarks of differentiated hCECs in the cornea and differentiated epithelial cells in the limbus [80]. In cultured epithelial tissue, KRT3/12 has been found in the suprabasal and superficial layers, but not in the basal layer of cells cultured on flat HAM, a finding corresponding to our results [79]. However, the KRT3/12 marker was reduced in the looped regions of the crypt-like HAMs. In some small looped regions, the respective marker was absent. The KRT3/12 marker is known to be absent from limbal basal layers—a finding that shows a more mature nature of the corneal basal cells compared to the limbal basal epithelial cells, due to different characteristics of the corresponding basal membranes [81]. Also, KRT3/12 is absent from limbal epithelial crypts [82].

Our study shows that unique cell fractions in the basal layers attached to the HAM are positive for the N-cadherin marker. It seems that the isolation of the cell cultures surrounded by a double HAM membrane increases cell positivity for this marker. N-cadherin is essential for maintaining the progenitor characteristics in cultured hLESCs [83]. Differentiated corneal and limbal epithelial cells express E-cadherin, while N-cadherin is present in the hLESCs/progenitor cells in the limbal basal epithelium, a finding concomitant with ours. In particular, our basal epithelial layer in the posterior limbus appeared to be enriched with this marker. It is suggested that communication with the melanocytes is achieved via N-cadherin forming homotypic adhesions [84].

A disadvantage of our technique may be that a larger area of HAM tissue is needed for transplantation and, evidently, a decreased transparency of the cultivated transplantation graft, in addition to any usual disadvantages of using HAM [26].

## 5. Conclusions

In conclusion, this novel HAM suturing technique increased the number of progenitor cells upon expansion and may thus increase the quality of the transplanted graft. We believe this technique can be a valuable, simple, and inexpensive tool to increase the success rate of corneal epithelial regeneration. However, the suturing technique needs to be tested in vivo to confirm its efficacy. Future clinical studies comparing conventional, flat suturing and the current suturing method are also required.

## Figures and Tables

**Figure 3:**
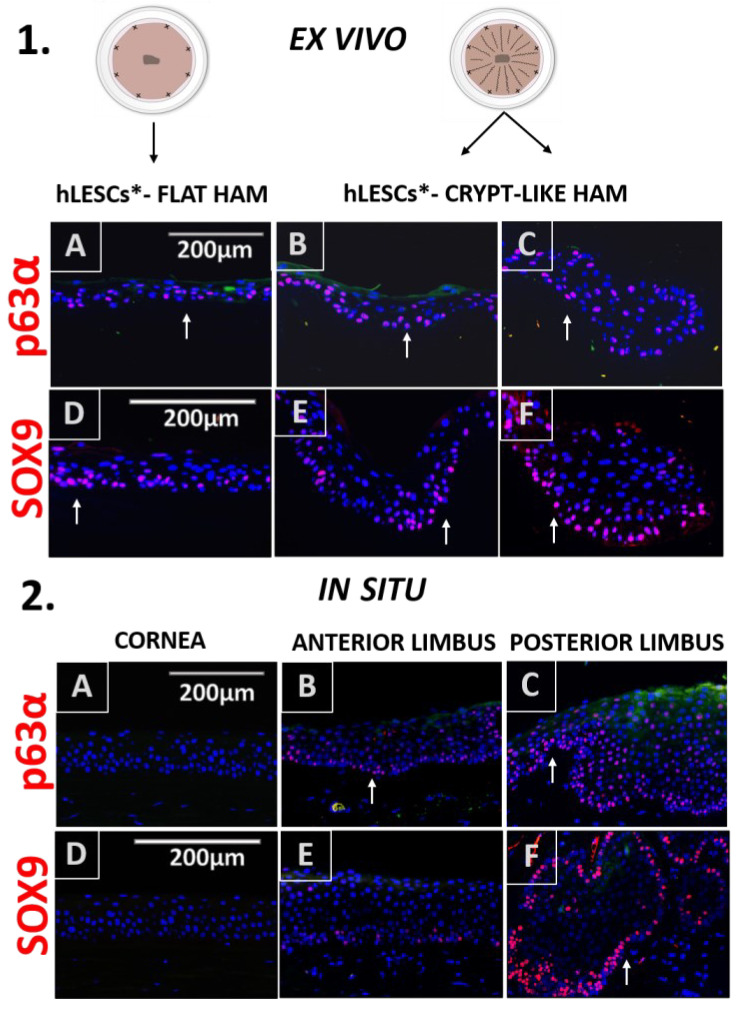
Progenitor marker distribution in situ in human epithelial corneal-limbal tissue and ex vivo in the hLESCs cultivated on the flat and crypt-like HAM structures. (1) The p63α and SOX9 presentation in hLESCs harvested on flat HAM ((1**A**,**D**) arrow) and crypt-like HAM structures: undulations ((1**B**,**E**), arrow) and loops ((1**C**,**F**), arrow). (2) The p63α and SOX9 marker expression in human corneal-limbal tissue: cornea ((2**A**,**D**)), anterior limbus ((2**B**,**E**), arrow), and posterior limbus and limbal epithelial crypts ((2**C**,**F**), arrow). In the corneal epithelium, p63α was absent. Scale bars are the same for all images (magnification: 40×); hLESCs*—human limbal epithelial stem cell cultures.

**Figure 4:**
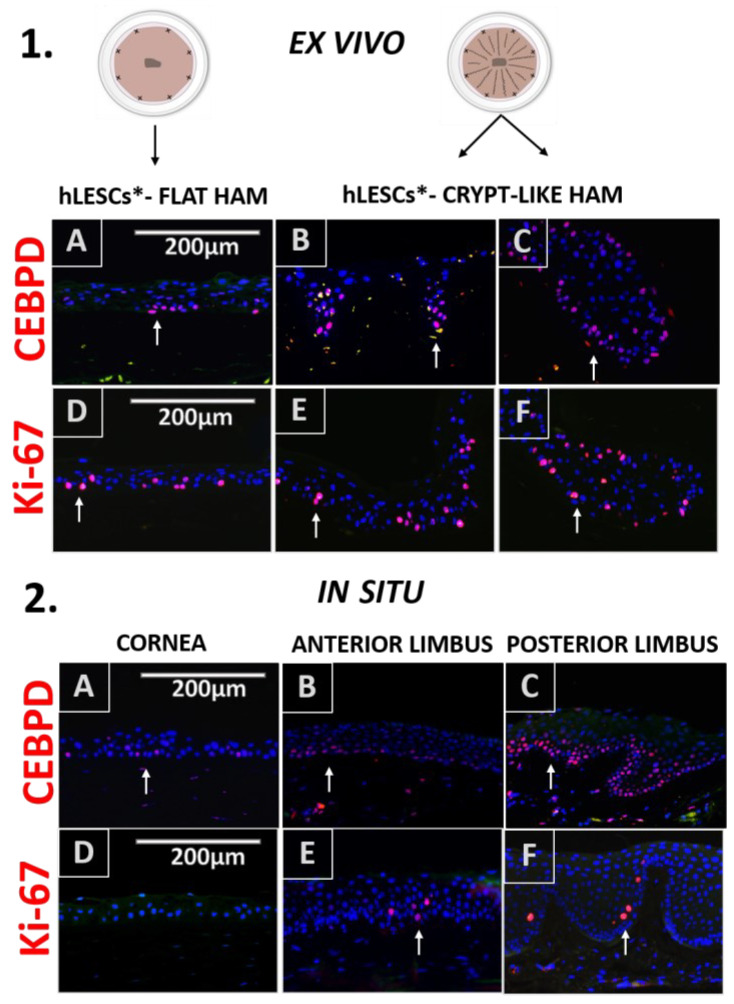
Quiescence and proliferation marker expression profile of in situ human epithelial corneal-limbal tissue and ex vivo hLESC cultures on flat and crypt-like HAMs. (1) CEBPD and Ki-67 were present in the basal and suprabasal layers of ex vivo hLESC cultures expanded on the flat (1**A**,**D**) and crypt-like HAMs. Novel suture technique generated undulated (1**B**,**E**) and looped (1**C**,**F**) HAMs containing hLESC cultures abundant for Ki-67- and CEBPD-positive cells. (2) Quiescence marker CEBPD and proliferation marker Ki-67 expression in the epithelium of cornea (2**A**,**D**), anterior (2**B**,**E**), and posterior (2**C**,**F**) limbus. Scale bars are the same for all images; hLESCs*—human limbal epithelial stem cell cultures.

**Figure 6:**
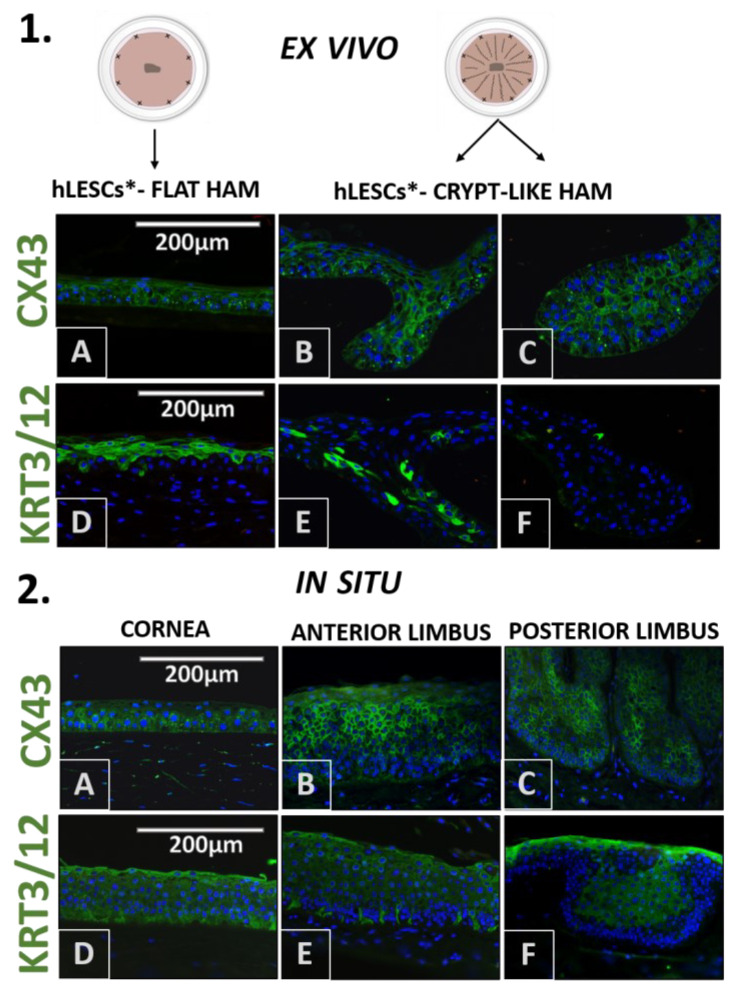
Differentiation marker expression profile of the ex vivo expanded hLESCs cultured on flat and crypt-like HAMs. (1) Polygonal and flat-like cells of the middle layers of cultures on flat HAMs stained positive for KRT3/12 antibody (1**A**,**D**). KRT3/12 was less present in hLESCs cultured on undulated (1**B**,**E**) and looped HAMs (1**C**,**F**). CX43 was present in most of the hLESC cultures, regardless of HAM formation (1**D**–**F**). (2) KRT3/12 and CX43 proteins were present throughout all cornea layers (2**A**,**D**), but they were absent in some cells of the basal limbal epithelium anteriorly (2**B**,**E**) and posteriorly (2**C**,**F**). Scale bars are the same for all images; hLESCs*—human limbal epithelial stem cell cultures.

**Figure 7:**
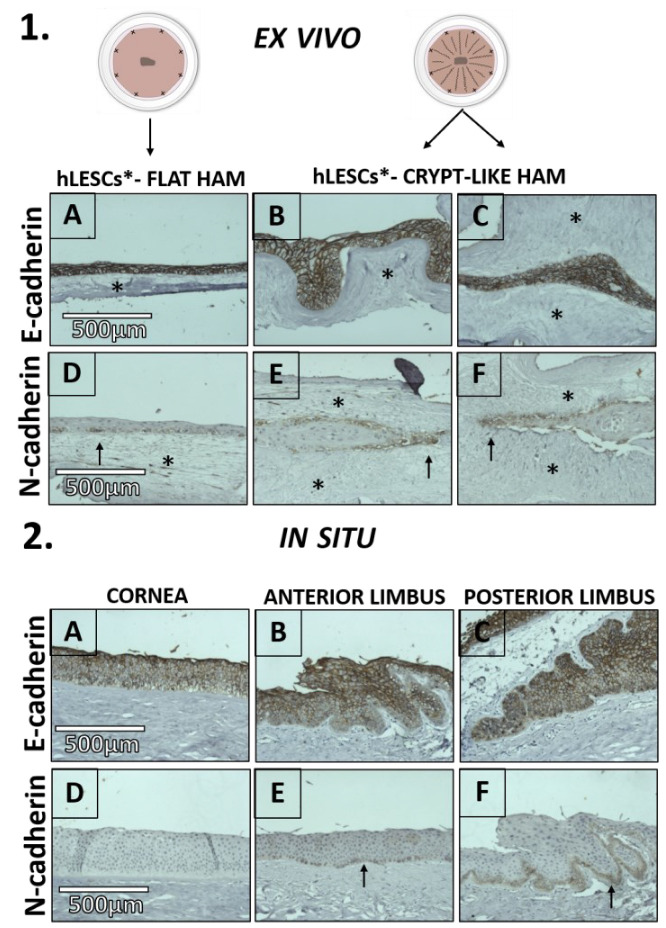
Transmembrane protein expression in situ in the epithelium of the corneal-limbal tissue and ex vivo expanded hLESC cultures on flat and crypt-like HAMs. E-cadherin presence in most cells of the hLESC cultures harvested on flat (1**A**), undulated (1**B**), and looped HAM (1**C**), as well as in the corneal (2**A**), and limbal (2**B**) epithelium and limbal epithelial crypts (2**C**). N-cadherin was present in some basal cells cultured on the flat HAM ((1**D**), arrow) and crypt-like HAMs ((1**E**,**F**), arrow). N-cadherin was absent in the corneal epithelium (2**D**), whereas it was present in cells of the basal limbal epithelium anteriorly ((2**E**), arrow) and posteriorly ((2**F**), arrow). HAM is marked with a black star. Scale bars are the same for all images in the same row.

## Data Availability

The data that support the findings of this study are available from the corresponding author upon reasonable request.

## Supplementary Materials

The following supporting information can be downloaded at: https://www.mdpi.com/article/10.3390/cells12050738/s1, Figure S1: The p63α and CEBPD marker staining of the expanded limbal epithelial tissue on the human amniotic membrane (HAM) ex vivo (1A–F) and corneal-limbal tissue in situ (2A–F); Figure S2: The Ki-67 and CEBPD marker staining of the expanded limbal epithelial tissue on the human amniotic membrane (HAM) ex vivo (1A–F) and corneal-limbal tissue in situ (2A–F); Figure S3: The CX43 and KRT/12 marker staining of the expanded limbal epithelial tissue on the human amniotic membrane (HAM) ex vivo (1A–F) and corneal-limbal tissue in situ (2A–F); Table S1: List of primary and secondary antibodies used for immunohistochemistry.
