# Supplementary material for: A Novel Technique of Amniotic Membrane Preparation Mimicking Limbal Epithelial Crypts Enhances the Number of Progenitor Cells upon Expansion
Source: Cells. 2023 Feb 24;12(5):738. doi: 10.3390/cells12050738 (PMC10001367; doi:10.3390/cells12050738)
Supplement: Supplementary file 1 [file cells-12-00738-s001.zip › cells-2195455-supplementary.pdf]

Supplementary information

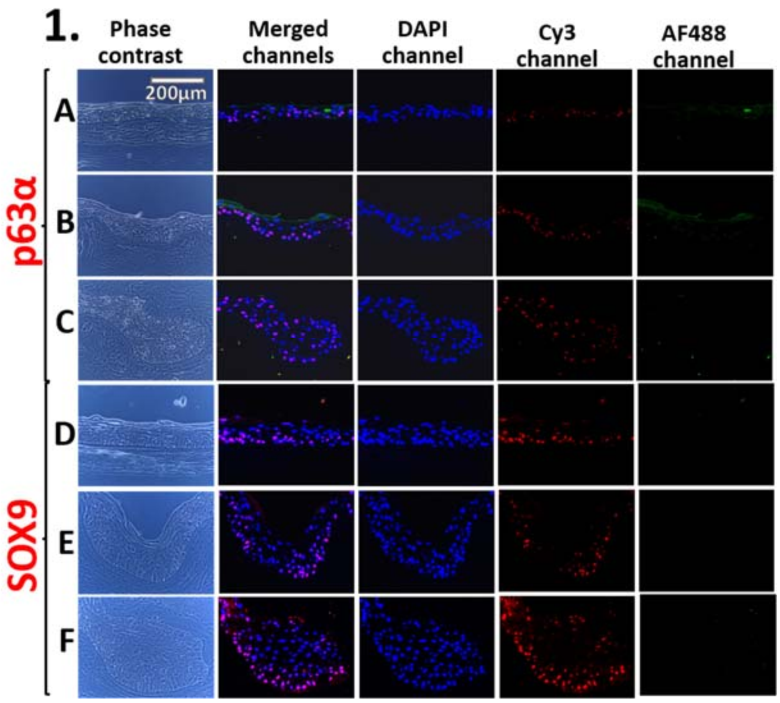

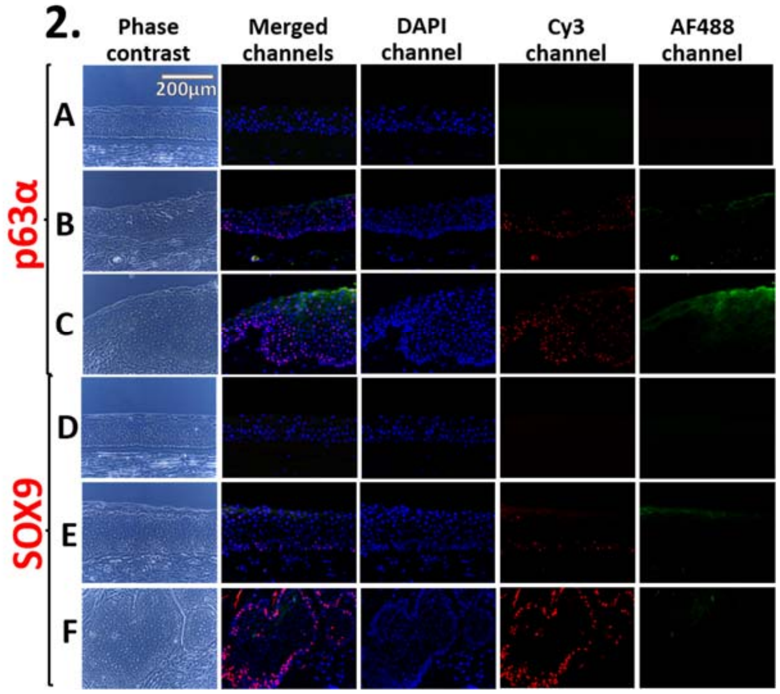

Figure S1. The p63α and SOX9 marker staining of the expanded limbal epithelial tissue on the human amniotic membrane (HAM) *ex vivo* (**1. A-F**) and corneal-limbal tissue *in situ* (**2. A-F**). Phase contrast (the first row to the left) images, as well as images of the merged and individual channels of the fluorescence immunohistochemistry (right) are presented.

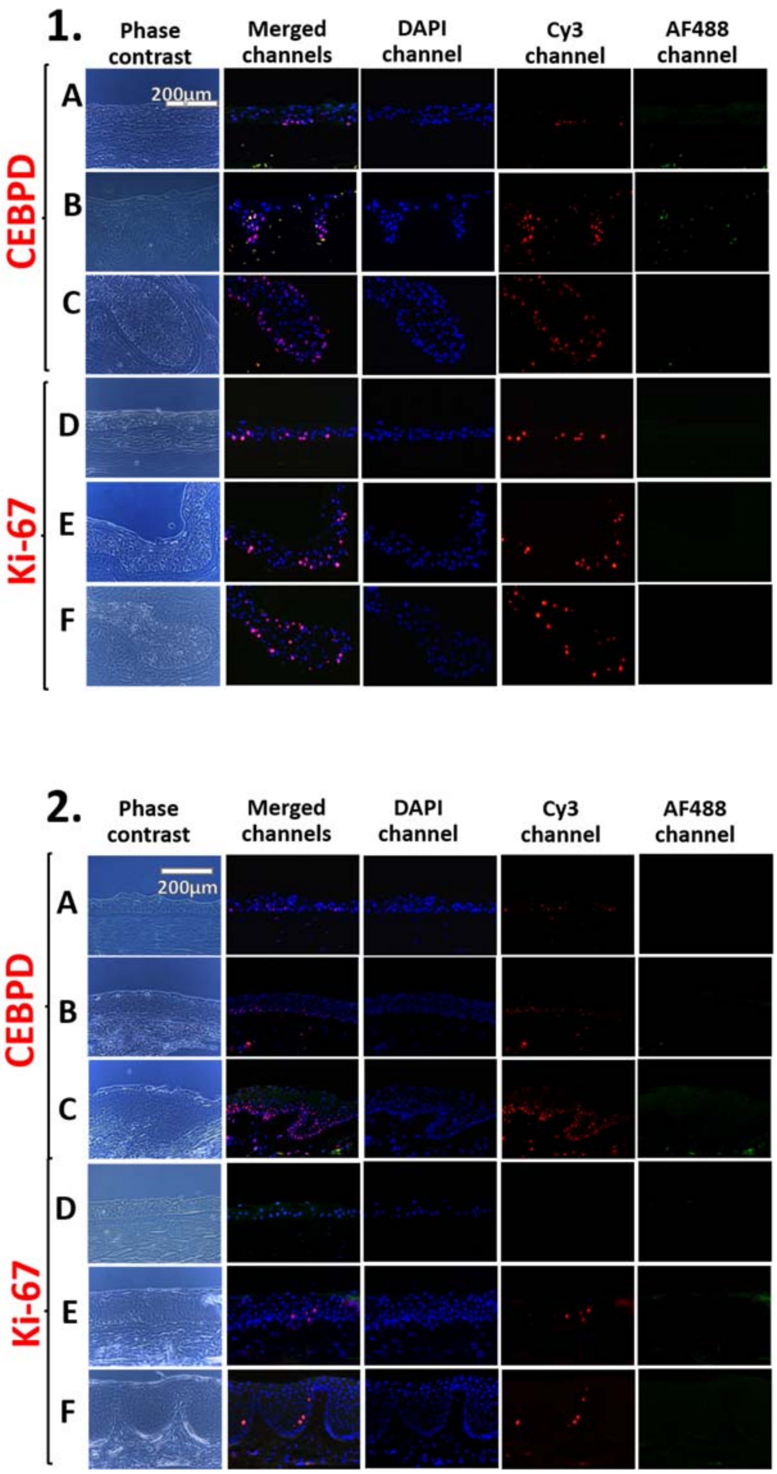

Figure S2. The CEBPD and Ki-67 marker staining of the expanded limbal epithelial tissue on the human amniotic membrane (HAM) *ex vivo* (**1. A-F**) and corneal-limbal tissue *in situ* (**2. A-F**). Phase contrast (the first row to the left) images, as well as images of the merged and individual channels of the fluorescence immunohistochemistry (right) are presented.

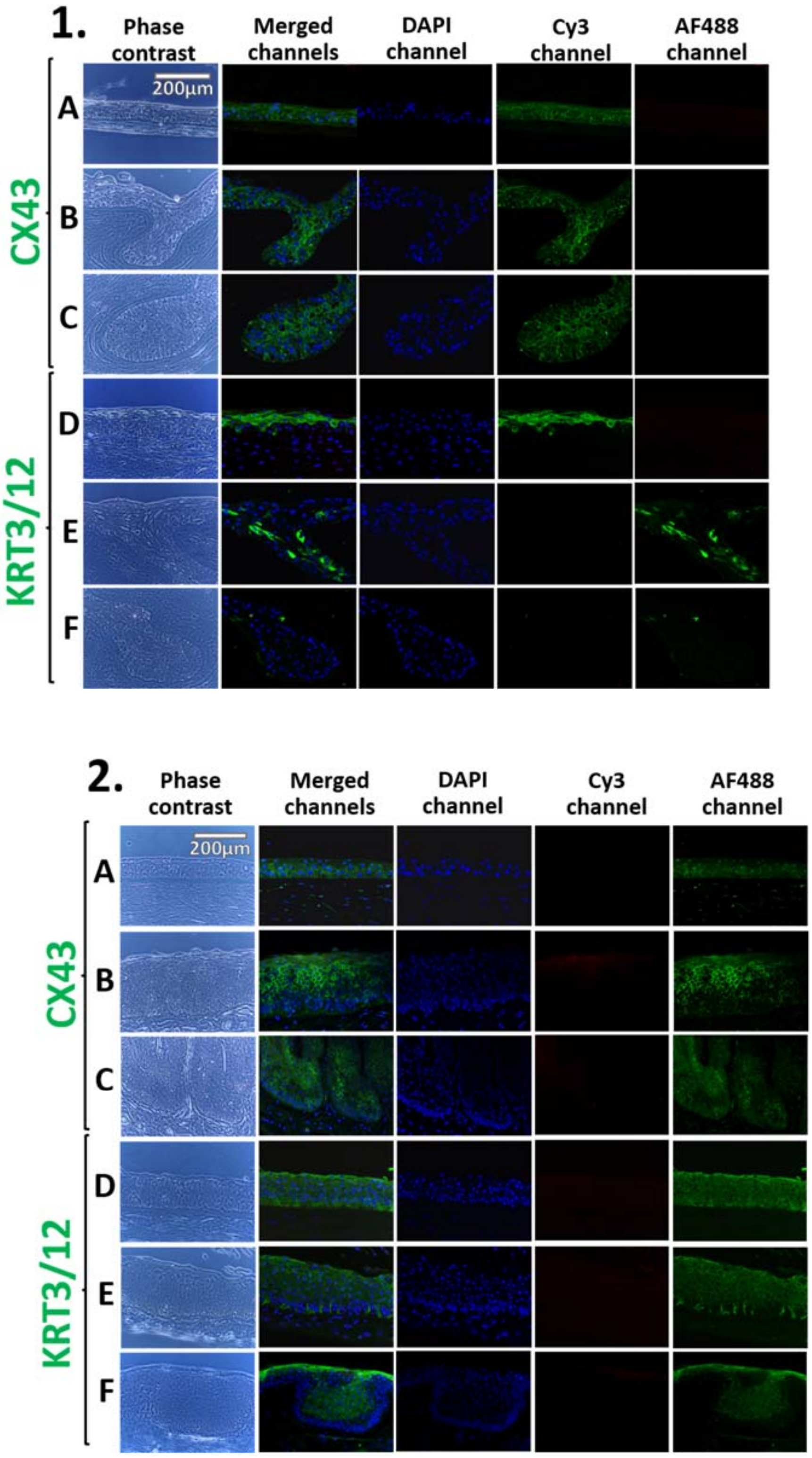

Figure S3. The CX43 and KRT3/12 marker staining of the expanded limbal epithelial tissue on the human amniotic membrane (HAM) *ex vivo* (**1. A-F**) and corneal-limbal tissue *in situ* (**2. A-F**). Phase contrast (the first row to the left) images, as well as images of the merged and individual channels of the fluorescence immunohistochemistry (right) are presented.

Table S1. List of primary and secondary antibodies used for immunohistochemistry

**Primary antibody**	**Symbol**	**Reference**	**Company**	**Host**	**Dilution**	**Clonality**
Tumor protein 63 alpha	p63α	4892S	Cell Signalling	Rabbit	1:200	Polyclonal
Proliferation marker Ki-67	Ki-67	RM-9106-S	ThermoScientific	Rabbit	1:200	Monoclonal
CCAAT/enhancer-binding protein delta	CEBPD	Ab198320	Abcam	Rabbit	1:200	Polyclonal
SRY-Box Transcription Factor 9	SOX9	82630	Cell Signalling	Rabbit	1: 200	Monoclonal
Connexin 43	Cx43	C6219	Sigma Aldrich	Rabbit	1: 1000	Polyclonal
Cytokeratin 3/12	KRT3/12	08691431	MP biomedicals	Mouse	1:100	Monoclonal
**Secondary antibody**	**Symbol**	**Reference**	**Company**	**Host**	**Dilution**	**Clonality**
Alexa Fluor® 488 donkey anti-mouseIgG		A21202	Abcam	Mouse	1:500	Monoclonal
Alexa Fluor® 488 donkey anti-rabbitIgG		A21206	Abcam	Rabbit	1:500	Monoclonal
Cy3® goat anti-rabbit IgG		A10520	Abcam	Rabbit	1:500	Monoclonal
